# Ectopic pregnancy with a contralateral corpus luteum: Case report

**DOI:** 10.5935/1518-0557.20220050

**Published:** 2023

**Authors:** Isaac Benjamin, José V. Figueira, Rodolfo Miquilarena, Francisco Rodriguez, Alia Lopez, Jorge Lerner

**Affiliations:** 1 Unidad de fertilidad. Clínica El Ávila. Caracas, Venezuela

**Keywords:** ectopic pregnancy, contralateral corpus luteum, ovum transmigration

## Abstract

The following report describes the case of an ectopic pregnancy with contralateral corpus luteum after spontaneous conception. The patient was a 33- year-old female (gravida 3, segmentary C sections 3), with positive pregnancy test, and an Intrauterine Device (IUD). The patient was asymptomatic. At vaginal ultrasound, we observed an anteverted uterus of normal shape and size, a 20 x 12 mm intramural myoma and an irregular endometrial thickness of 16.5 mm, with no intrauterine sac. An ectopic pregnancy in the left Fallopian tube and a contralateral corpus luteum were detected, possibly as consequence of ovum pick up through the opposite tube (oocyte transmigration). Further laparoscopic and histopathologic studies confirmed our findings, and the ectopic pregnancy was successfully removed. In conclusion, oocyte transmigration is a common event and should be account when we wish to provide medical advice to patients with a single Fallopian tube trying to conceive. There are real chances for a patient to become pregnant even when only a single tube is present.

## INTRODUCTION

The mechanism through which the oocyte is captured by the Fallopian tube and its subsequent transport remains uncertain. The conventional anatomical representation of the ovaries, tubes and uterus, would indicate that the capture of the oocyte from the surface of the ovary is produced by the ipsilateral tube; however, in vivo anatomy, allows us to understand the possibility that the oocyte is extruded by the ovary into the peritoneal fluid where it could be captured by either of the two Fallopian tubes (Ross *et al*., 2013).

The event in which the pregnancy occurs contralateral to where ovulation was produced, is called ovum transmigration or transperitoneal migration, and the clinical opportunities to record it are limited: through visualization of an ectopic pregnancy with a contralateral corpus luteum, or in pregnancies (ectopic or intrauterine) in patients with a single tube and a corpus luteum in the opposite ovary.

In this study, we present the report of a patient with an IUD under Isotretinoin treatment, who presented an ectopic pregnancy in the left tube, and a corpus luteum in the right ovary.

## CASE DESCRIPTION

The following report is undertaken with the approval of the medical society of “Clínica El Ávila” Medical Center in Caracas, Venezuela. It refers to a female patient, 33 years old with history of gravida 3, para 3 (all segmentary C-Sections).

The patient has been using a T-Cu 380A (Copper IUD) since January 2015, and referred use of Isotretinoin (Roacutan^®^), since February 2021, for acne treatment. The patient attended the gynecologist on June 11, 2021 due to a 5-week evolution amenorrhea and a beta human chorionic gonadotropin (β-hCG) quantitative test of 800 mU/ml. Isotretinoin was suspended and T-Cu 380A was removed.

The patient attended our office for a second opinion on June 14, 2021. The patient had regular menstrual cycles occurring every 28 days and lasting approximately 5 days, the LMP (last menstrual period) was on May 5, 2021. Asymptomatic, on physical examination: portrayed good general conditions, afebrile to touch, hydrated, conscious, space, time, and person oriented, normotensive, with normal and rhythmic pulse and normal respiration, depressible soft non-painful abdomen, no visceromegaly. At the transvaginal ultrasound, we observed an anteverted uterus of normal shape and size, heterogeneous myometrium, a 20 x 12 mm intramural myoma on anterior wall and an irregular thick endometrium of 16.5 mm. Right ovary showed a cyst image of 25 mm, corresponding to a corpus luteum. Left ovary was normal in size and echo pattern. Quantitative β-hCG was indicated, and a value of 4555 mU/ml was obtained.

She was evaluated again by way of an ultrasound on June 16, 2021, evidencing an 8.3 x 6.6 mm gestational sac, a 3.7 x 4.0 mm yolk sac with no visible embryo, at left para uterine level. Given the diagnosis of an ectopic pregnancy with right corpus luteum, treatment alternatives were given: a complete blood count, a metabolic panel and a quantitative β-hCG test, prior to medical treatment with a single dose of 100 mg of intramuscular Methotrexate. A quantitative β-hCG 4 days after treatment was also requested.

Before the medical treatment, the β-hCG test on June 16, 2021 was 9031 mU/ml. Due to increasing β-hCG values, the patient was warned of the possibility of associated symptomatology based on the results. On June 20, 2021, a transvaginal pelvic ultrasound was performed, with the following findings: at left para-uterine level, a 12.3 x 10.9mm gestational sac was observed, with a 4.7 x 5.0mm yolk sac and visible embryo. Crown Rump Length (CRL): 1.7 mm and cardiac activity with bradycardia. β-hCG: 22780 mU/ml.

**Figure 1:**
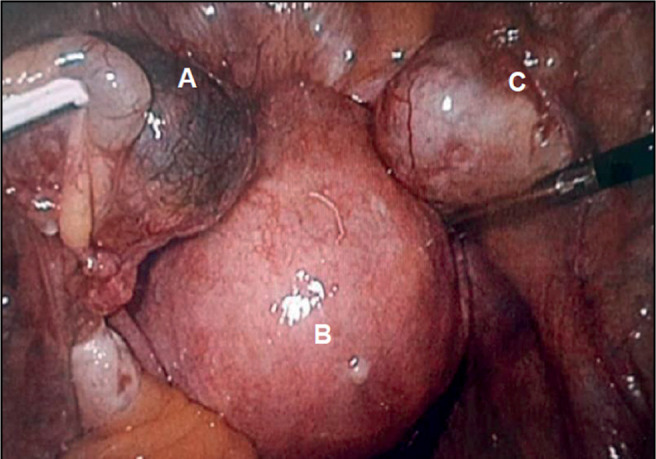
Laparoscopic view of pelvis. A = Left fallopian tube with ectopic pregnancy. B = Uterus. C = Right ovary with corpus luteum.

On June 21, a surgical laparoscopy was suggested and performed, with the following results (Figure 1): a normal size and shape uterus, with a normal right Fallopian tube. The right ovary was increased in size due to an approximately 25 mm cyst, compatible with a corpus luteum. The left Fallopian tube was increased in size and presented an irregular shape due to a 20 mm tumor, corresponding to a gestational sac. Left ovary was normal.

Left salpingectomy was performed with no complications. Patient evolved satisfactorily and was discharged the following day.

Patient reported menstruation two days after the intervention. β-hCG control test on June 23, 2021 was 2436 mU/ml. β-hCG values were monitored until a value of less than 5 mU/ml was observed.

**Figure 2:**
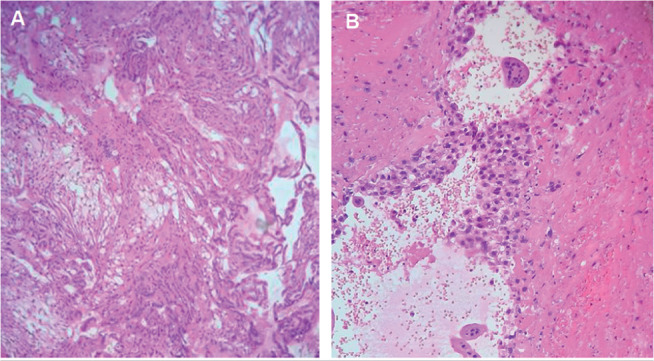
Histopathology study from the left tube. A = Fragment chorionic villi (H&E x 100). B = Trophoblast cells (H&E x 100).

In the histopathology study (Figure 2), the left tube tissue displayed compatible findings with an ectopic pregnancy, recrudescence chronic salpingitis, para tube serous cyst. No cell atypia was observed.

## DISCUSSION

An ectopic pregnancy is defined as a pregnancy outside of the uterine cavity, diagnosed by ultrasound, surgical or histopathological visualization (Zegers-Hochschild *et al*., 2017). It is consequence of the implantation of the blastocyst outside of the uterine cavity, representing 1 to 2% of the pregnancies in the United States and Europe, but reaches a 75% of maternal deaths during the first trimester and a 9 to 13% of all maternal deaths (Creanga *et al*., 2011; Varma & Gupta, 2009). In developing countries, mortality rates are up to ten times higher than in industrialized nations (Goyaux *et al*., 2003).

Due to its possible complications, it is crucial to carry out an early diagnosis, by means of a high index of clinical suspicion and two complementary studies: the transvaginal ultrasound and the detection of β-hCG hormone in serum (Montgomery *et al*., 2017).

The risk factors for an ectopic pregnancy include: current smoking habits, a recent history of consumption of ≥ 10 g of alcohol per day, exposure to diethylstilbestrol in the uterus, use of oral contraceptives at an early age, history of infertility, pelvic inflammatory disease, infection due to Chlamydia trachomatis, IUD use (as is the case of our patient) and tubal ligation (Gaskins *et al*., 2018).

In this case report, our patient presented a tubal pregnancy contralateral to the corpus luteum, which could be explained through ovum transmigration. When carrying out a coronal cut of the uterus, by means of the transvaginal pelvic ultrasound to explain the pelvic anatomy; the tubes and the ovaries are located in extension, which leads us to think that the tube generates a sweep over the ipsilateral ovary at the time of ovulation. However, the truth is that the tubes are located posterior to the uterus, at the bottom of the pelvic sac and beneath the ovaries, which allows us to understand the theory of the peritoneal sweep by any of the two Fallopian tubes.

Some publications (Berlind, 1960; Berry *et al*., 1985; Insunza *et al*., 1988) proposed the ovum transmigration as a possible etiology for ectopic pregnancy. In a review in 2002, Ziel & Paulson (2002) reported that the event of an ectopic pregnancy contralateral to the corpus luteum had an incidence of 15 to 60% (Berlind, 1960; Berry *et al*., 1985; Breen, 1970; Honoré, 1978; Kleiner & Roberts, 1967; Insunza *et al*., 1988; Saito *et al*., 1975; Walters *et al*., 1987; Wheeler & Dodson, 1985; Ziel & Paulson, 2002), which was a presumed consequence of ovum transmigration. As the hatching of the blastocyst is a programmed event, a delay in the arrival of the embryo to the uterus (product of oocyte transmigration), would predispose the occurrence of an ectopic pregnancy. However, more recent studies (Nogueira *et al*., 2011; Ross *et al*., 2013) confirm that oocyte transmigration is a frequent event, with an incidence of 32% in both intrauterine and ectopic pregnancy, in patients with a single tube.

Moreover, Tubal Embryo Transfer (TET) has not demonstrated higher ectopic pregnancy rates than the Gamete Intra-Fallopian Transfer (GIFT); therefore, it is unlikely that the delayed arrival of the gametes to the site of fecundation would be the cause for an ectopic pregnancy (Balmaceda *et al*., 1988; Craft & Brinsden, 1989; Ziel & Paulson, 2002).

As for the occurrence of an ectopic pregnancy due to embryo migration through the uterus, its incidence is still unknown. Intrauterine embryo migration is described in In Vitro Fertilization (IVF), so there is a chance that this rare event could occur spontaneously (DiLuigi *et al*., 2008). And as for the event of patients with an intrauterine pregnancy and both tubes, the incidence of oocyte transmigration is speculative (Nogueira *et al*., 2011).

In this case report, our patient had an IUD, which increases the risk of an ectopic pregnancy. Additionally, the patient was in treatment with Isotretinoin. This medication has a potent teratogenic effect, increasing the risk of cardiac, cranioencephalic and central nervous system anomalies (Henry *et al*., 2016); and its use must be avoided during pregnancy, or should be prescribed together with some contraceptive method (as in the case of our patient), but with no success. A possible relation between the use of Isotretinoin and the etiology of the ectopic pregnancy has not been identified (Mitchell *et al*., 1995).

Being a young patient, conservative treatment is always preferable. Although, the patient had expressed no desire of wanting more children. We decided to start with the Methotrexate, being a safe medical alternative for the treatment of an unruptured ectopic pregnancy and no embryo, in order to avoid invasive surgeries with possible complications (Mittal *et al*., 2003). Given the rise of β-hCG hormone, and the evidence on transvaginal ultrasound of a gestational sac in the left tube with an embryo and cardiac activity; laparoscopy surgery was considered, corroborating the findings raised in the clinic.

In conclusion, this is our first experience documenting an ectopic pregnancy with contralateral corpus luteum, presumably produced by oocyte transmigration. Although this event seems to be more common than expected, we certainly do not think about this mechanism when a spontaneous pregnancy occurs; or when we wish to provide medical advice to patients with a single Fallopian tube trying to conceive. The clinical opportunities to record a contralateral pregnancy to the corpus luteum are scarce; therefore, we consider it a remarkable event to present.
